# Efficacy of Virtual Reality Distraction in Reduction of Pain and Anxiety of Pediatric Dental Patients in an Iranian Population: A Split-Mouth Randomized Crossover Clinical Trial

**DOI:** 10.1155/2024/1290410

**Published:** 2024-01-12

**Authors:** Zahra Bahrololoomi, Javad Zein Al-Din, Nahid Maghsoudi, Samira Sajedi

**Affiliations:** ^1^Department of Pediatrics, School of Dentistry, Shahid Sadoughi University of Medical Sciences, Yazd, Iran; ^2^Department of Pediatrics, School of Dentistry, Shahrekord University of Medical Sciences, Shahrekord, Iran

## Abstract

**Materials and Methods:**

This crossover clinical trial was conducted with eligible 6–8-year-old children requiring bilateral mandibular molar pulpotomy. At the first treatment visit, pulpotomy was performed for 15 children using VR glasses distraction while the other 15 children received a pulpotomy without any VR glasses; this trend was reversed at the second session and pulpotomy was performed for the contralateral tooth. Pulse rate (PR) and Modified Child Dental Anxiety Scale (MCDAS) measured the anxiety levels. Wong–Baker Faces Pain Scale (WBFP) assessed the pain perception before and after the intervention. Data were analyzed by Statistical Package for the Social Sciences version 25 using the Mann–Whitney and *χ*^2^ tests.

**Results:**

The mean PR was not significantly different between the two groups. However, the test group showed significantly lower scores of MCDAS (*P* value = 0.02) and WBFP (*P* value = 0.001) compared with the control group.

**Conclusion:**

The present results suggest that VR headsets can decrease the level of pain and anxiety of patients during primary mandibular pulpotomy. This trial is registered with IRCT20200315046782N1.

## 1. Introduction

Dental visits are challenging, especially for dental-phobic patients [1]. Negative dental office experiences, especially due to dental pain, often have consequences such as increased levels of fear and anxiety, poor cooperation of children, dissatisfaction of parents, and development of a negative attitude toward dentistry. Dental anxiety has a strong and significant relationship with poor oral health-related quality of life. Children with high dental anxiety are also shown to have decreased emotional well-being due to their oral health status [2–4].

The prevalence of dental fear has been reported 13.3%–36.5%, in preschoolers, schoolchildren, and adolescents [5, 6]. Children with higher levels of dental anxiety often have more serious dental complications and a lower frequency of dental visits compared with other children, and it leads to worse dental visit behaviors and lower Frankl's Behavior Rating Scale (FBRS) score [7, 8]. Thus, behavioral guidance techniques along with effective local anesthesia should be administered for painless dentistry to decrease the pain and anxiety of children and promote a positive perception of dentistry in children [7, 9].

Distraction is a safe and affordable strategy for behavioral guidance, which draws away an individual's attention from painful stimuli and thereby reduces the reception of related information [10]. The attention capacity of human beings is limited, and focusing on painful stimuli leads to increased pain perception. Thus, the level of pain can be decreased by distraction [11]. Distraction can be performed by watching TV, listening to music, counting the objects in the operatory room, and having nonmedical conversations with the child to ease the process of treatment [7, 10]. Distraction develops cognitive conditions including passive coping skills which the child copes without directly encountering the stressful situation [12]. In recent years, great attention has been directed to virtual reality (VR) for behavioral guidance. VR was first introduced for entertainment. However, it has been widely applied in different clinical fields such as pain control and treatment of psychological conditions in the past 10 years [13, 14]. VR is an advanced technology for creating a virtual environment in which, the patient can actively interact at different levels with different senses [15].

In contrast to audiovisual devices, VR uses more complex systems that involve 3D monitors with an extensive field of view in the form of a headset and movement monitoring systems that measure the movements of the head and hands. Since a VR headset does not allow the child to see the actual stimuli in the operatory room, it fully distracts the child and focuses his/her attention on the virtual world. Thus, it has a significant superiority over other types of distraction and the child will be immersed in the environment [16, 17].

VR has been the subject of investigation in several studies, including its use in different dental procedures [11, 18, 19]. However, the results have not been consistent and comprehensive enough to draw a definitive conclusion. Czech et al. [20] have also reported on the need for more meticulous studies due to limited research in this field and too much heterogeneous data to be pooled as a meta-analysis. Therefore, this study aims to assess the effect of distraction through VR on the level of pain and anxiety experienced by 6–8-year-old children during primary molar pulpotomy.

## 2. Materials and Methods

### 2.1. Study Design

This split-mouth randomized crossover clinical trial was approved by the ethics committee of Yazd University of Medical Sciences (IR.SSU.REC. 1398.097) and registered in the Iranian Registry of Clinical Trials (IRCTID: IRCT20200315046782N1).

### 2.2. Sample Size Calculation

The sample size was calculated at a 5% level of significance and 80% study power. Finally, the minimum sample size was found to be 30 patients.

### 2.3. Participants, Eligibility Criteria, and Settings

The inclusion criteria were (I) children between 6–8 years old without any history of previous dental care seeking bilateral primary mandibular molar pulpotomy, (II) physically and mentally healthy children who were ASA (I) or (II), (III) children presenting score 3 or 4 of FBRS [21], (IV) no history of severe toothache or dental or medical emergencies, and (V) no history of anxiety disorders, children with the scores <25 of the Screen for Child Anxiety Related Disorders (SCARED) questionnaire were recruited. SCARED evaluate the likelihood of trait anxiety in child and discriminates between depression and anxiety [20]. A written informed consent was signed by the child's parents only after a complete explanation of study procedures.

The exclusion criteria were (I) uncooperative children, (II) not showing up for appointments, (III) necrotic tooth, (IV) uncooperative parents, and (V) use of analgesics before treatments.

### 2.4. Interventions

At the examination visit, the SCARED questionnaire assessed the presence of anxiety disorders such as separation or generalized anxiety disorders, panic disorders, and school avoidance in children. A total score higher than 25 may indicate the presence of an anxiety disorder in the child; such children were not enrolled in the study.

All study procedures were performed by a third-year pediatric dentistry resident. Complete intraoral examination was performed for all children at the examination session and they all received fluoride therapy. Dental instruments were introduced to them by the Tell Show Do technique and the child's cooperation was evaluated based on FBRS [21]. Finally, the radiographs were administered and the next visit was scheduled 1 week later. Each child required bilateral pulpotomy and a simple random list determined which side would be treated at the first visit. Another random list was used to choose whether VR glasses would be used at the first or second session for each patient.

Large VR glasses (LEJI VR Mini glasses, China) cover a major part of the face which can cause anxiety by blocking the child's vision of what happening around him/her. Thus, a mini glass has been chosen for the present study. The VR headset was placed on the patient's face and showed Tom and Jerry animation through an Apple iPhone 6 (Apple Inc, Beijing, China) connected to the VR headset.

Group A: A pulpotomy and restorative treatment without VR glasses was performed at the first visit. Topical anesthesia (20% benzocaine; Medental International Inc., USA) was first applied on dried mucosa followed by an inferior alveolar nerve block of 2% lidocaine with 1 : 80,000 epinephrine (Persocaine, DarouPakhsh, Iran). A 27-gauge needle with 35 mm length was used for all patients in both visits. For pulpotomy, first, the caries were removed by a low-speed handpiece (NSK-Nakanishi, Japan), and then the access cavity was created by a high-speed handpiece (NSK-Nakanishi, Japan). The pulp status was clinically evaluated and in the case of necrosis (presence of puss or no bleeding), the patient would be excluded from the study. In case to achieve optimal hemostasis of the pulp, irrigation with saline was performed, a cotton pellet dipped in formocresol (Master-Dent, USA) was placed over the pulp for 5 min, and after fixation of the pulp, Zonalin paste (Kemdent, UK) was applied over it. The tooth was restored with a stainless steel crown (3M ESPE, USA) at the same session. A week later patient returned for the treatment of the contralateral tooth and VR glass was used during local anesthesia and pulp therapy.

Group B: The VR headset was first introduced to the child by the Tell Show Do technique at the first visit. It was then placed on the child's face and the animation was started. Topical anesthesia and inferior alveolar nerve block were administered, and pulpotomy was performed as explained for Group A. The second treatment session was scheduled 1 week later while the VR headset was not applied during pulp therapy.

Factors such as the assistant, operatory room, working environment, and conversations with the dentist were the same for all children in both sessions. The duration of the procedure was approximately 30 min. The Modified Child Dental Anxiety Scale (MCDAS) was filled out before and after the procedure and the Wong–Baker Faces Pain Scale (WBFP) was also used to quantify the level of pain experienced by the children immediately after treatment was done. The pulse rate (PR) as a physiological parameter was measured by a digital pulse oximeter (Buerer PO 30; Germany) before and after the procedure.

The validity and reliability of MCDAS have been previously confirmed. This questionnaire has eight questions that assess the level of anxiety of the child using a 5-point scale. Thus, the maximum and minimum total scores would be 40 and 8, respectively. Scores <19 indicate no anxiety, scores >19 indicate the presence of anxiety, and scores >31 indicate severe phobic disorder [22].

The WBFP is a simple comprehensive self-report scale for pain assessment, which uses pictorial measures to quantify the level of pain. Each question is scored 0–10 by selecting one of the five faces with different expressions from a happy face to a crying face.

### 2.5. Randomization

The participants were randomly assigned into two groups using a 38-item list created by a statistician. Both groups were generated through simple randomization. The first list indicated the allocation of the patient to Group A or B at the first session: (a) no headset and (b) using the headset. The second list determined which side will be treated at the first pulpotomy visit: (1) right quadrant and (2) left quadrant. Sealed opaque envelopes in two colors were used for randomization. The sequence of the first list was entered into blue and the second list was entered into red envelopes. All envelopes were placed in a box and shuffled entirely, each patient was requested to draw one red and one blue envelope after enrollment and obtaining written informed consent from the parents. The envelopes were opened and the content indicated the using/not using of the headset in the first treatment session and the quadrant to undergo treatment [23].

### 2.6. Blinding

Blinding was not possible in this study.

### 2.7. Statistical Analysis

Data were analyzed using Statistical Package for the Social Sciences version 25. The Kolmogorov–Smirnov test was used to assess the normality of data distribution. Collected data of PR, WBFPS, and MCDAS scores showed abnormal distribution; thus, the Mann–Whitney test was applied to analyze these data. The *χ*^2^-test was used to compare gender between the two groups.

## 3. Results

**Figure 1:**
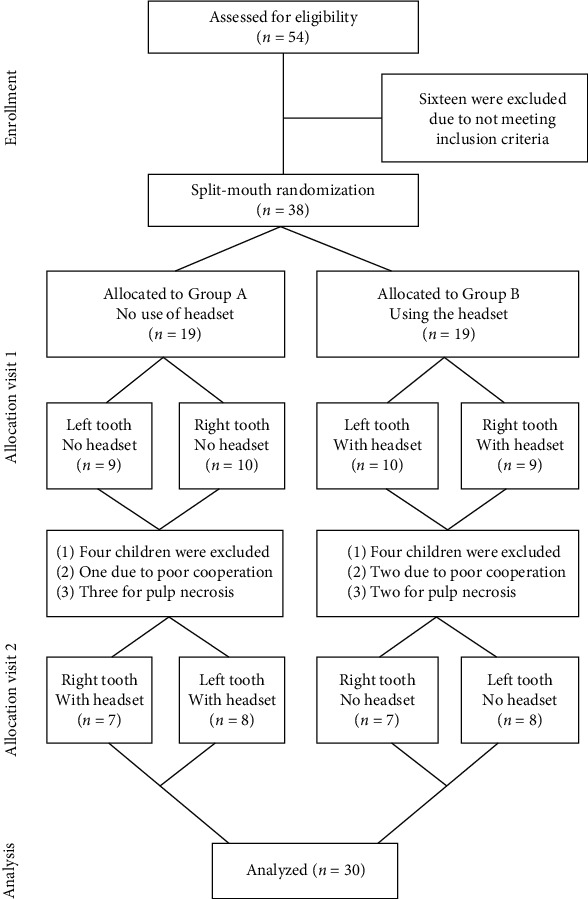
Flowchart of the trial.

The present study was performed with the children referring to the Pediatric Dentistry Department of Shahid Sadoughi University of Medical Sciences between October 2019 to February 2020. Fifty-four children were screened and 38 children were recruited to the study; in the course of treatment, eight children (four from the test and four from the control group) were excluded. Finally, 30 children (15 controls and 15 test children) who required bilateral pulpotomy of primary mandibular molars were evaluated (Figure 1).

A number of 14 (46.7%) male and 16 (53.3%) female patients completed the study. The *χ*^2^-test showed no significant differences in gender distribution (*P* value = 0.642).

The Mann–Whitney test showed no significant difference in PR before and after the procedure in the two groups (*P*=0.89, Table 1).

The mean scores of MCDAS (*P* value = 0.02) and WBFPS (*P* value = 0.001) were significantly lower in the test group compared to the control group (Tables 2 and 3).

## 4. Discussion

This study aimed to assess the effect of distraction by VR on pain and anxiety perception in 6–8-year-old children requiring bilateral primary molar pulpotomy. Pain and anxiety perception are impressed by different parameters such as personality, gender, and family factors. Crossover design has been chosen to eliminate the effect of such confounding factors and interindividual differences, each patient served as his/her own control in two treatment sessions [11]. Prior unpleasant dental experiences may trigger anxiety and fear in forthcoming dental visits so children without any history of dental treatments who showed a score of 3 or 4 on the FBRS have been recruited [3]. Gender distribution was 46% male and 53% female to compensate for the effect of gender differences on the results.

The term “presence” indicates how much the user is immersed in the virtual environment which has a great significance in VR distraction. Changing position, orientation, point of view, and field of view affect the immersion. Also, passive audiovisual devices with cartoon videos create less immersion resulting in less distraction and more pain perception. Thus, a VR glass has been chosen to distract the patients to achieve profound immersion [16].

The results showed lower levels of pain and anxiety in the test group; these differences were significant for anxiety measured by MCDAS and self-reported pain (WBFPS) but not significant for PR. MCDAS and WBFP scales are self-report tools for assessing pain and anxiety which are more preferred in the literature. The WBFP scale has been chosen as the best rating method by the nurses and all age groups of children. It provides adequate psychometric properties and excellent validity [24]. MCDAS subjectively measures the anxiety focusing on the trait anxiety which is stable and persistent. While heart rate acts as a psychological and objective tool, it measures the anxiety as a response to the stressful situation of a dental visit called state anxiety which is related to the momentary mood [17].

The 1-week washout period between dental appointments was intentionally set by the clinicians intending to prevent any misinterpreting observations about study-related treatments that were actually due to prior appointment. We wanted to ensure that all children had an equal gap between their appointments while also taking into account the limitations of the dental center. After careful consideration, we determined that a 1-week washout period was the most appropriate and achievable option.

It has been observed that children under the age of 6 do not have enough cognitive development to accurately rate their pain levels. In fact, they tend to overstate their pain levels, which can lead to an incorrect interpretation of their self-reported pain scales [25]. To ensure that we get valid results, it was crucial to select a group of children who have similar cognitive and behavioral development. Therefore, for this trial, we enrolled children between the ages of 6 and 8 years old.

The present results were in agreement with those of El-Sharkawi et al. [26] and Al Khotani et al. [18]. El-Sharkawi et al. [26] assessed pain perception during the inferior alveolar block in clinically designed studies on children. Reported subjective and objective pain levels were lower when audiovisual distraction was used compared with control groups [26]. Al Khotani et al. [18] also showed lower anxiety levels and more cooperative behavior using audiovisual distraction during dental restoration treatments. El-Sharkawi et al. [26] and Al Khotani et al. [18] have used regular videotaped cartoons which provide less involvement than 3D videos nevertheless they showed the efficacy of audiovisual glasses as a distraction method. The possible explanation may be less invasion of performed treatments in the mentioned studies compared with the present trial [18, 26].

The present results were in line with those of Aminabadi et al. [11], they used VR glasses to distract 4–6 years old children during restorative treatment and significant decreases were observed in pain and anxiety of the test group compared with the control one.

Buldur and Candan [27] conducted a study in Turkey to evaluate the impact of VR on children's dental anxiety, pain, and behavior. The trial involved children between the ages of 7 and 11, and composite restoration was performed on mandibular molars under anesthesia. According to heart rate scores, the VR group showed a significant reduction in dental pain and anxiety [27]. Similarly, Al Khotani et al. [18] conducted a clinical trial in Riyadh, Saudi Arabia, to investigate the effect of audiovisual distraction on the behavior of children aged 7–9 years during dental treatment. Their results showed lower dental anxiety in the audiovisual group, and there was a significantly lower PR during the injection of local anesthesia [18]. Mitrakul et al. [28] also researched the effect of audiovisual eyeglasses during dental treatment in Tai children aged 5–8 years old. Their results indicated a reduced PR and physical distress during preoperation and the first use of a high-speed handpiece [28].

In their study on a Spanish population, Gómez-Polo et al. [29] found that using a VR headset during dental treatments significantly reduced anxiety levels (95% of the children were happy) and improved behavior (100% positive behavior) compared to the control group (40% and 57.5%, respectively). The researchers utilized the Facial Image Scale Test and FBRS to evaluate anxiety levels and the child's behavior of the children during their first and last appointments [29].

Koticha et al. [19] reported different results, which may be due to the different assessments of anxiety levels. They used the Venham Picture Test to measure anxiety levels which is not quite clear, the flashcards and figures are ambiguous in meaning for children. The MCDAS scale which was used in the current study has shown good internal consistency and validity and it has increased rates of full completion in comparison to the other scales such as the Child Fear Survey Schedule—Dental Subscale (CFSS-DS). Also, Koticha et al. [19] and Holmes and Girdler [30] evaluated patients who required tooth extraction, which is a more stressful procedure than pulpotomy. In a crossover trial, authors did not show any significant differences between video eyeglasses and traditional nonaversive behavior management. Dental treatments were not equalized in the test and control groups. Also, pain was only measured objectively (Face, Leg, Activity, Cry, and Consolability (FLACC) scale), while subjective measurement of pain has been reported as a more accurate assessment tool [19].

In general, as shown in the present study, VR headsets can be used as an effective distraction tool and improve the behavioral management of children. However, factors such as the type of equipment used, the animation, the level of interest of the child in the VR headset, the expertise of the dental clinician, and the communication skills of the child can all affect the results.

This study had some limitations. The participants of the study were selected by convenience sampling from a single treatment center, and many environmental factors that could influence their dental anxiety and pain were overlooked. To ensure the validity of the findings, future studies should be conducted in multiple centers with larger sample sizes. The study's results might be affected by the relatively small sample size, as well as the lack of consideration for the cultural, economic, and educational background of parents and their parenting style. Additionally, the child's background and attitude toward VR glasses could also have an impact on the results.

The headsets used in the experiment were primarily designed for use in a seated or standing position. As a result, patients in a supine position on a dental unit may experience discomfort when the headsets are used during the treatment of maxillary teeth. This should be taken into account when considering the use of these headsets in dental procedures. Another limitation of the study was that all participants were provided with the same audiovisual material. However, previous research has highlighted the importance of allowing participants to select their own audiovisual content and how it can impact their behavior. Allowing children to choose their own audiovisual material can increase their comfort with the VR device and lead to longer usage times, as well as giving them a sense of control that can help reduce stress levels. In clinical dental settings, children often have limited control over their surroundings, so giving them control over the content they view can be beneficial [27]. Distraction with VR glasses could be used for younger children as they are often more involved in magical thinking and are truly attracted by imaginary plays. Thus, as a suggestion, this intervention could be performed on younger samples [31].

## 5. Conclusion

Within the limitations of this study, considering the safety and availability of VR headsets and the fact that their use requires no prior instruction, they can be used for the reduction of pain and anxiety in children.

## Figures and Tables

**Table 1:** Mean value of pulse rate.

	Group	*N*	Mean	Standard deviation	*P* value
PR before treatment	Control	30	95.53	9.35	0.640
	Test	30	92.80	12.20	

PR after treatment	Control	30	96.80	10.23	0.561
	Test	30	93.80	10.78	

The mean difference in PR	Control	30	1.73	2.40	0.890
	Test	30	−0.87	5.11	

**Table 2:** Mean MCDAS score.

	Group	*N*	Mean	Standard deviation	*P* value
Anxiety before treatment	Control	30	10.87	2.10	0.312
	Test	30	10.46	2.32	

Anxiety after treatment	Control	30	10.66	2.61	0.281
	Test	30	9.90	2.67	

The mean difference in MCDAS score	Control	30	1.00	1.13	0.022
	Test	30	−0.6	1.99	

**Table 3:** Mean of WBFP score.

	Group	*N*	Mean	Standard deviation	*P* value
WBFP score before treatment	Control	30	1.06	1.49	0.025
	Test	30	0.27	0.70	

WBFP score after treatment	Control	30	2.13	1.76	0.004
	Test	30	0.93	1.48	

The mean difference in WBFP score	Control	30	0.93	1.49	0.001
	Test	30	−1.20	1.47	

## Data Availability

The data that support the findings of this study are generated during the study and are available from the corresponding author.
